# Round the Hospitals

**Published:** 1918-03-16

**Authors:** 


### ROUND THE HOSPITALS.

A meeting of the members of the College of Nursing residing in Liverpool and the locality will be held in the Lecture Theatre of the Royal Infirmary, Liverpool, on Monday, March 25, at 3 p.m. If any members are unable to attend they are requested to send their names and addresses to the honorary secretary of the Liverpool Centre, Miss Worsley, the Children's Infirmary, Liverpool.

Previous articles appeared Jan. 19, p. 337; 26, p. 357; and Feb. 9, p. 401; 23, p. 441.

**ROUND THE HOSPITALS—(*continued*).**

Miss M. A. Willcox has been appointed matron of King's College Hospital, where she was trained from 1905 to 1908, becoming ward sister in that year at the Royal Free Hospital, a post which she held for eighteen months. From 1910 to 1914 she was at the Surgical Home at Manchester, when she joined the Territorial Force Nursing Service, and in August 1914 was sent to the 4th London General Hospital. In May 1915 she was appointed charge sister at No. 15 General Hospital, Alexandria, and in October 1915 matron of the hospital for officers in the Sirdar's residence at Cairo. She returned to England in October 1916, since when she has been assistant matron at the Ruskin Park Extension of the 4th London General Hospital. Miss Willcox has had a remarkably active and diversified experience in hospital work. The rapidity and variety of the changes which she has courageously faced and successfully lived up to since she joined the Territorial Force Nursing Service on the outbreak of war mark her as a matron with many gifts who should prove a tower of strength as the head of her old training-school, where a hearty welcome awaits her.

---

The memorial service for nurses who have died on duty or from injury sustained during the war has been arranged by the Dean and Chapter to take place at St. Paul's Cathedral on April 10, at 2.30. Queen Alexandra has expressed her intention of being present if possible. The sermon will be preached by the Ven. E. E. Holmes, B.D., Archdeacon of London. Space in the Cathedral will be reserved for nurses in uniform and for the relatives and friends of the fallen. Full directions as to the centres to which applications for seats should be made by members of the various branches of the nursing services are given on page 516 of this issue. The Roll of Honour is being compiled by the Rev. A. Lombardini, St. Elizabeth's Church, Kensington Infirmary, Marloes Road, London, W., and comprises already nearly a hundred names from different branches of the Nursing Service, naval and military. We hope to be able to publish later fuller particulars relating to those for whom this service of thanksgiving is to be held. The blood of martyrs is the seed of the Church, and modern historians now recognise more and more clearly the debt which Christianity owes to those who laid down their lives for the truth. May it not be that the life-blood of the nurses, freely surrendered at the call of duty to succour their brothers, may have in it a saving grace to lift their profession and, indeed, spur all women on to attain a higher level of service for humanity?

---

The Episodes in the History of St. Bartholomew's Hospital, by Lt.-Col. D'Arcy Power, F.S.A., which appeared in that hospital's journal last month, contain much delightful matter tending to throw light on the hours, remuneration, and duties of the nurses in old days. Under date 1565 we learn of the lay sisters and nurses being set under a matron, instead of under the Mother Superior. "They came not out of the ward every night after the hour of seven o'clock in the winter and after nine o'clock in the summer except some great and special cause befell—as the present danger of death or the needful succour of some poor person. They washed and purged the unclean clothes of the patients and other things and in their spare time they were set to spinning the flax." . . . "Above all things they were told to abhor and detest scolding as a most pestilent and filthy vice." In 1747 we find the matron entitled to "an old and accustomed fee" of one shilling for the use of a pall to cover the coffin of every patient buried from the hospital, the sisters claiming a shilling and the nurses sixpence from patients and their friends "for earthenware and other necessaries." In 1821 there were 24 wards nursed by 24 sisters, 48 nurses, and 26 night nurses. The salaries of the sisters ranged from 14s. to 27s. a week, whilst the nurses received 7s. a week, and the night nurses 9d. a night.

---

The end of Col. D'Arcy Power's retrospect is worth recording: "I have a vision of a time when the present Nurses' Home shall have been swept away and in its place there has arisen in Little Britain a fine building with a good lounge, a pleasant drawing-room, a well-equipped library, fine baths, plenty of hot and cold water, a separate little bedroom for each, a lift for tired nurses, and an infirmary on the topmost floor made as little like a hospital ward as possible. Such a building has long been overdue, but it must come, for our present arrangements are disgraceful and are a standing reproach to the great city of which we have formed an integral part for nearly a thousand years." Alas, the war has rendered building and reconstruction operations impracticable perhaps for years to come, and it will not soon be forgiven to the hospital authorities that during the period of unexampled prosperity which preceded the war they took no steps whatever to wipe out the reproach which has rested on them so long.

---

In refreshing contrast to the apathy displayed by the governors of St. Bartholomew's Hospital in regard to their "disgraceful" Nurses' Home, is the action of the trustees of the Liverpool Royal Infirmary. Undaunted by the embargo on building, and with sublime confidence in the public generosity where the well-being of nurses is concerned, the trustees have lately opened a fund for the erection of a new Nurses' Home for the Royal Infirmary so soon as the Government may permit such necessary works to be begun. Undoubtedly this is one of the most noted provincial training-schools for nurses in the country. Over sixty nurses from this establishment have already given their services to the country in the war. Its usefulness to the public and value to the nursing profession will be greatly enhanced when it is able to offer the nurses accommodation worthy of their profession. We foresee immediate and sustained support for the movement initiated to provide the new Nurses' Home.

**ROUND THE HOSPITALS—(*continued*).**

The outstanding feature of the Report of the Committee of Management to the members and nurses on the staff of the Nurses' Co-operation is the continued maintenance of the staff at full strength in an abnormal time. A large number of the nurses have been engaged in nursing soldiers, and every endeavour has been made to meet the calls made by the War Office and Admiralty. During the last year one of the military hospitals in England was entirely staffed by the Co-operation Nurses. The Royal Red Cross has been awarded to Sisters Babb, Baxter, J. C. Bell, Chaplin, S. Gardner, Leech, McGill, Mucklow, Paul, Ashlin, Thomas, and Young. Including thirty-two asylum-trained nurses and twenty-seven nurses on six months' probation, the staff now numbers 522 nurses, who receive the whole of their earnings, subject in the case of those who joined before the end of 1908 to a deduction of 5 per cent., and in the case of those admitted since of 7½ per cent. The founding, under Miss Philippa Hicks, of this historic Co-operation for Nurses, the first in which nurses' earnings were secured to them, was alluded to as an epoch in nursing by Mr. P. Michelli, C.M.G., secretary of the Seamen's Hospital, at the recent meeting on behalf of the College of Nursing, held at the Dreadnought Hospital.

---

THE Ministry of National Service is intervening to help the Metropolitan Asylums Board over their impasse in regard to probationers. There has been tremendous competition for young women, and the hospitals were bound to suffer. The infectious hospitals and sanatoria under the Board have been more hardly hit than any, and a serious epidemic of any kind necessitating an immediate large increase of staff might land them in serious difficulties. As it is, new and badly needed wards cannot be opened for lack of nurses. As many as 700 untrained women are required, we learn, some as probationers, some as ward-maids and for other domestic duties. It is a class of nursing which for obvious reasons lies outside the scope of volunteer helpers, but the conditions of service are not wanting in attraction, and we believe that much might be done locally to popularise the service. A leaflet, attractively written, clearly setting out the rate of pay, hours, and prospects of promotion, with photographs of the wards and nurses' quarters, would open the eyes of the public and bring in plenty of candidates. And, as we said last week, the salaries ought to be revised.

---

THE appointment of Miss Beatrice Monk, A.R.R.C., chief assistant of Miss Lückes, to the post of steward at the London Hospital during the enforced absence of the present steward, who is in ill-health, is an event of the first importance in connection with the movement for training women to undertake the commissariat. The office which Mr. Hills has so ably held for twenty-five years is responsible to a degree little imagined by the outsider. It demands integrity of no common order, such as carries with it the power to enforce clean dealing on every individual admitted to transactions with the hospital. The knowledge needed for the purchase and control of stores for this immense establishment is extensive and peculiar at all times. At the present moment the problems and obstacles to success may be considered as double those in ordinary times. Ten years ago there were hardly any women who had received a training to fit them for undertaking the higher posts relating to the commissariat, and the appointment of a woman steward to a major institution would have been deemed fantastic and foredoomed to failure. The London, in common with one or two other large general hospitals, has now for some time afforded admirable training in the higher branches of housekeeping to its sisters, and the extension of this training will doubtless be facilitated by Miss Monk's appointment. Under her we may hope to see a succession of women stewards trained for similar posts.

---

THE new Army regulations relating to members of Voluntary Aid Detachments affect a very large number of useful people, and have been received with a chorus of approval from the "assistant-nurses," their friends and relations, and last, but not least, from the matrons and sisters under whom they have been serving. Such as have already won the red efficiency stripe for the grade of V.A.D. assistant-nurse are now to be entitled to wear a badge "A.N." in metal on their aprons for indoor uniform and on the shoulder-straps of their outdoor uniform coat. The majority of those thus qualified have had practical and theoretical training in the wards not so unlike what the regular probationer receives, though of course severely limited in scope. Many matrons of military hospitals take a keen interest in the instruction of their V.A.D. assistants, give them lectures, institute examinations for them, and stimulate them to master the theory and practice of nursing by every means in their power. The new regulations will strengthen the hands of these far-sighted matrons by setting a coveted distinction to be attained before the eyes of their pupils. Promotion of the special military probationers and V.A.D. nursing members to the new grade is to depend on efficiency, not on mere length of service. In practice we imagine it will be distinctly awkward to inform candidates for the distinction, after two years' hard work in the wards, that they have been efficient enough to be retained in the service of the hospital but not to wear the letters "A.N." on their apron.

---

MRS. EDITH PHILLIPS, matron of the great Saidieh Red Cross Hospital at Giza, in Egypt, writing to Sir Arthur Stanley after a tour of inspection, reports that there are twelve sisters and a matron at Jerusalem, all getting comfortably

**ROUND THE HOSPITALS—(*continued*).**

settled after "quite a war front time," and that the Red Cross Advance Store has arrived by motor-lorry over the mountains. There had been heavy fighting, evidenced by the numerous little crosses on the mountains, those on the Mount of Olives being "wonderfully impressive." Red Cross stores and comforts have been supplied to the wounded of the Sheriffian army at Hedjaz, where there is a British medical officer in charge, and also to a number of French medical units in the Red Cross area. None of the campaigns has been followed with so much interest and sympathy by the public as this, and every woman whose nursing duties take her to Palestine in these days may be considered a privileged person; a category which does not, however, exclude a more than common measure of hardships to be endured.

---

The City and County Nursing Association fills a place at Worcester of more than ordinary importance, since it embraces and co-ordinates nursing not only in the town but in all the outlying districts. The extent to which local authorities now depend on the services of benevolent nursing associations is illustrated by the fact that the Local Government Board paid £165, the Educational Committee of the County Council £100, and the Insurance Committee £150 for services rendered. Fifty nurses have left the Association to take up war nursing, some of those on the staff have undertaken the nursing of sick soldiers at Norton Barracks, and help has been given in lecturing to and examining candidates for the Voluntary Aid Detachments. It is abundantly evident that the work which has fallen to the nurses, particularly in midwifery and child welfare, is increasingly arduous, and Dr. Dixey was on sure ground when he stated at the annual meeting that the salaries ought to be substantially increased also. Public grants certainly ease the finances of the nursing societies, and this ought to be reflected in the nurses' pay.

---

Children at Cromwell House, Highgate Hill, convalescing tubercular cases from Great Ormond Street Children's Hospital, are making remarkable progress in one item of their carefully adapted schooling. Cursive writing, hitherto generally taught to London school children on "commercial" lines, a sloping character with looped consonants and large capitals, has now been replaced by the so-called "manuscript," in which every letter is made separately and as much like the printed character as possible. The result is noteworthy, and bears out the testimony given to the style by Binet and others. Relieved of the continuous tension and of the special difficulty of the long "fine" upstrokes, the child's hand gains confidence and strength, and in an extraordinarily short time excellent writing on one line is produced by children of six to nine. Moreover, the word, being better visualised, is more easily spelt. Such difficult words as "February" and "eight" were conquered at once. For the child who learns to write while lying on his back in bed the merits of the system are immense; those who have once seen the results will wish it introduced into every institution where physically handicapped children are trained.

---

The Halifax disaster, the full horror of which is only now beginning to be understood in this country, has exposed the need for some organisation, says the *Canadian Nurse*, similar to the American Red Cross, which will be all equipped and ready for mobilisation at a moment's notice. The Canadian Red Cross, like the British, with which it is affiliated, is limited in its scope to soldiers and wounded prisoners. Something on the lines of the St. John Ambulance Society would seem to be indicated, ready to undertake service wherever required and enlisting civilian help. One of the most painful features of the Halifax disaster is the number of totally and partially blind sufferers from the flames and the intense cold of the blizzard.

---

☞ It is our invariable practice to pay for all items of news which we may publish. The minimum payment is 5s. Paragraphs of about twenty lines in length—that is to say, of 150 words or less—are preferred. Contributions should be addressed to The Editor, The Hospital, 28 and 29 Southampton Street, Strand, London, W.C. 2, and, to be in time for the current week's issue, should reach him by the first post on Mondays.

